# Vaccination in pregnancy against pertussis and seasonal influenza: key learnings and components from high-performing vaccine programmes in three countries: the United Kingdom, the United States and Spain

**DOI:** 10.1186/s12889-021-12198-2

**Published:** 2021-11-29

**Authors:** Théophile Baïssas, Florence Boisnard, Inmaculada Cuesta Esteve, Marta Garcia Sánchez, Christine E. Jones, Thierry Rigoine de Fougerolles, Litjen Tan, Olivier Vitoux, Christina Klein

**Affiliations:** 1 CVA, London, UK; 2 Sanofi Pasteur, 14, Espace Henry Vallée, 69007 Lyon, France; 3 Asociación Nacional de Enfermería y Vacunas, Zaragoza, Spain; 4 Hospital Quirónsalud Málaga, Málaga, Spain; 5 Faculty of Medicine and Institute for Life Sciences University of Southampton and NIHR Southampton Clinical Research Facility and Biomedical Research Centre, University Hospital Southampton NHS Foundation Trust, Southampton, UK; 6 CVA, Paris, France; 7 Immunisation Action Coalition, Saint Paul, MN USA

**Keywords:** Immunisation, Influenza, Maternal, Pregnancy, Prenatal, Pertussis, Tdap, Vaccine coverage rate

## Abstract

**Background:**

Pertussis and seasonal influenza are responsible for significant maternal, neonatal, and infant morbidity and mortality, but vaccine coverage rates (VCR) for both pertussis (administered as a tetanus, diphtheria, acellular pertussis [Tdap] vaccination) and seasonal influenza in pregnancy remain generally low. Only a small number of countries, including Spain, the United Kingdom (UK), and the United States (US), have high Tdap and seasonal influenza VCRs in pregnancy. The purpose of this study was to identify the key factors that contributed to the high VCRs observed in these countries.

**Methods:**

The experience from both Tdap and seasonal influenza vaccination programmes during pregnancy were documented in Spain, the UK, and the US using a three-step approach. A literature review yielded 157 publications, and a further 117 documents were selected through desk research. A published five-pillar VCR framework for influenza was amended to evaluate the specific contributing factors leading to high Tdap and seasonal influenza VCRs among pregnant women.

**Results:**

The analysis identified components that contributed to higher VCR in pregnant women across three different healthcare systems in Spain, UK, and US. The combination of several key interventions in each country led to a rapid increase in VCR that reached near-optimal levels (i.e. 75% for seasonal influenza) within a few years. As well as inclusion in national immunisation programme and vaccine reimbursement, key components that were identified included the mobilisation of health authorities, prenatal care Healthcare Professionals (HCP) and scientific societies, the inclusion of vaccination in antenatal medical guidance, the provision of educational material to HCPs, and a strong disease awareness driven by recent pertussis outbreaks in each country.

**Conclusions:**

Although there is no simple, universal solution to improving sub-optimal VCRs, the list of components identified in this study from three countries with high-performing Tdap and seasonal influenza vaccination programmes provides a basis for public health and medical stakeholders in other countries to define strategies to successfully implement national vaccination programmes for pregnant women.

**Supplementary Information:**

The online version contains supplementary material available at 10.1186/s12889-021-12198-2.

## Background

Vaccination against seasonal influenza and pertussis in pregnancy has proven effective in reducing the burden of seasonal influenza among pregnant women and their infants as well as reducing the severity of pertussis in young infants [1–4]. Pregnant women and children particularly those less than 6 months of age are considered to be priority risk groups for influenza since they experience increased rates of outpatient visits, hospitalizations, and deaths [5, 6]. However, influenza vaccination is only recommended from 6 months of age and therefore cannot provide protection to the youngest, most vulnerable infants. Even though the evidence on the burden of pertussis in vulnerable adults is growing, severe pertussis infection mostly affects infants between birth and 6 months of age [7, 8]. Infants under 6 months of age accounted for 42.3% of all pertussis-related hospitalizations in 2018 among children in the United States (US) [9]. A modelling study by the World Health Organization (WHO) highlighted that there were 85,900 estimated pertussis-related deaths in infants younger than 1 year in 2014 worldwide [10]. With no pertussis vaccines licensed to protect new-borns in their first weeks of life, pertussis vaccination in pregnancy remains the most effective method of providing protection during this vulnerable period.

In 2005, the WHO recommended influenza vaccination for all pregnant women. Due to the severity of 2009 H1N1 pandemic infections among pregnant women, this group was recommended as the highest priority group for inclusion in influenza immunisation programmes [11, 12]. This not only provides protection of the mother from severe disease but also protects the infant in the first months of life, before they are eligible to receive influenza vaccination. However, while influenza vaccination during pregnancy was gradually adopted in most high- and middle-income countries, many low-income countries have yet to include it in their routine immunisation programmes [13–17]. Furthermore, many existing programmes reach an influenza vaccine coverage rate (VCR) that is sub-optimal compared with the WHO target rate of 75% for the elderly and other risk groups [18–21]. In nine EU/EEA Member States, VCR ranged from 0.5 to 59% (median 25%) in 2016–17. The remaining EU/EEA Member States, where influenza vaccination is recommended for pregnant women, reported that vaccination coverage was not monitored for this population [22].

In 2015, the WHO recommended pertussis vaccination in pregnancy as the most cost-effective additional strategy for preventing disease in infants too young to be vaccinated [23]. The routine use of combination pertussis, diphtheria, and tetanus vaccines (Tdap) means that maternal immunity is boosted against these infections in addition to the provision of passive neonatal protection. Currently, pertussis vaccination during pregnancy is recommended by the national or supranational health authorities in more than 55 countries globally [24, 25]. Yet, despite growing adoption and funding under national immunisation programmes, many countries consistently fail to achieve among pregnant women a VCR as high as in childhood vaccination programmes. Only a few high-income countries successfully vaccinate a majority of pregnant women, such as Spain, the United Kingdom (UK), or the US with respective VCRs of 84%, 71%, and 57% in 2019 [26–29].

The WHO has developed several tools to support countries considering the introduction of pregnancy vaccination programmes or to improve the implementation of existing programmes. These include a toolkit for Influenza Vaccine Post-Introduction Evaluations and a dedicated field guide for the implementation of pregnancy vaccination in Latin America [30, 31]. However, actionable information for a successful programme of vaccination in pregnancy remains limited.

This study aimed to provide a thorough analysis of the programmatic components that contribute to the success of influenza and Tdap vaccination programmes in pregnancy based on practices in high-income countries achieving high VCR.

## Methods

### Methodological basis

A range of methodologies were considered to analyse the performance of influenza and Tdap vaccination programmes in pregnancy. From WHO models on vaccine hesitancy to practical taxonomies for the determinants of vaccine uptake such as the 5As (Access, Affordability, Awareness, Acceptance, Activation), several methodologies have been developed to comprehend the outcome of vaccination programmes [32, 33]. Kassianos et al. study was selected as a methodological backbone for this study given its emphasis on identifying actionable programmatic and policy components contributing to high seasonal influenza VCR amongst high-income countries with adult vaccination programmes [34]. Based on an analysis of the vaccination programmes in the US, UK, Canada, and Australia, this methodological framework consists of five pillars structuring 42 components as contributing factors of high VCR among older adults, some of which are also relevant to vaccination in pregnancy. Since the factors contributing to vaccination in pregnancy may differ significantly from those among older adults, and since our study also includes Tdap vaccination, the five-pillar structure and list of components from Kassianos et al. was adapted and expanded based on data gathered in three relevant benchmark countries (Spain, UK, and US). A three-step data collection approach was thus used in the considered countries including a detailed review of published literature using an academic literature database, a manual search of official sources, and a complementary search of grey literature.

### Benchmark country selection

For this analysis focusing on vaccination in pregnancy in high-income countries, Spain, the UK and the US were selected due to their pioneering approach to establishing both influenza and pertussis pregnancy vaccination programmes, their success in improving uptake, and the availability of annual VCR measurements by their respective health authorities to analyse the components that contributed to VCR growth. Even though other high-income countries than the three considered have developed pregnancy vaccination programmes they did not fulfil these three selection criteria and were thus not selected for this study. Furthermore, the US, with a mostly privatized and fragmented healthcare system, the UK with a public and centralized system, and Spain with a public and decentralized system were chosen as three illustrative examples to encompass different health system architectures. Since VCR values are published for England and Wales in separate reports, only England values are considered in this study to analyse trends given that the nation holds 84% of the UK population compared to 5% for Wales [35].

### Data collection

The data collection relied both on global and country-specific published literature, and a manual search of official sources as well as grey literature available online (Table 1**)**. An Embase search was performed to identify relevant published articles. Search terms were refined through Emtree subject headings searches and structured in three categories: Disease, Vaccination, and Population. Disease search terms in each category are detailed in Additional file 1 (Table SI 1). The search was performed for each country and publications from 2010 to 2020 were considered. All the titles and abstracts identified through these searches were screened using a patient, intervention, comparison, outcome (PICO) search strategy (Table SI 2), followed by a screening of the full text. As a result, 86 articles were selected for the US, 33 articles for the UK, and 23 articles for Spain (Table 1).

**Table 1:** Sources of information utilized in the data collection

	United States of America	United Kingdom	Spain	Total
**Review of published literature using Embase**				
Embase search yield	1252	834	117	2203
Selected articles	86	33	23	192
**Manual search of official sources**				
Health authority reports	23	14	18	55
**Complementary search of grey literature**				
Healthcare professional & lay public communication material	22	16	17	55
Market reports	2	1	3	6
Others	0	1	0	1

Additional manual searches of health authority (HA) websites and reports, leading scientific societies and research groups position papers, healthcare professional (HCP) associations training documentation, lay public communication material, and key conferences were conducted to limit potential publication bias in this study (Table 1). Such sources were also used to identify official VCR data and to evaluate the measures and actions implemented over time to improve VCR.

### Elaboration of the pregnancy VCR framework

Based on the published literature, official documents and communication materials identified in the three countries considered, all components described as contributing to the performance of pregnancy vaccination programmes were listed. Since VCR is a multifactorial variable and as the impact of some components may take time to materialize, the components were not selected purely on the direct quantitative impact on VCR, but rather based on whether they were described as a key contributor of higher vaccine uptake among pregnant women. The framework was consequently adapted to vaccination in pregnancy by matching identified components with those of Kassianos et al. framework to either add, edit or replace them in the relevant pillars.

## Results

**Fig. 1:**
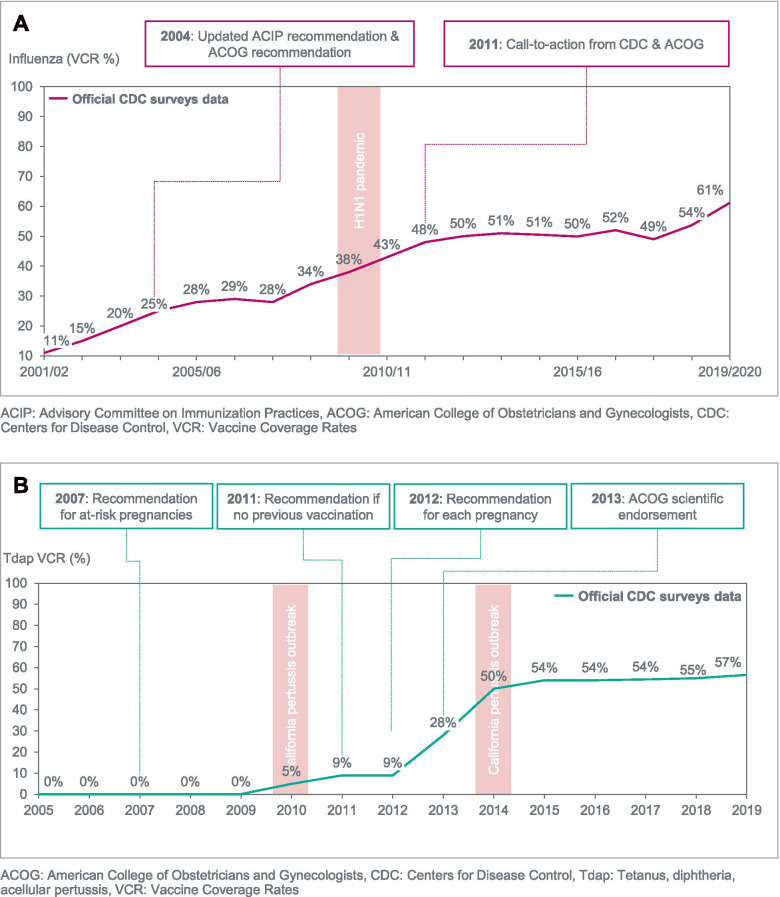
Evolution of VCR for influenza (2001–2019) and Tdap (2005–2019) among pregnant women in the US. **A** Influenza. **B** Tetanus, diphtheria, acellular pertussis (Tdap)

**Fig. 2:**
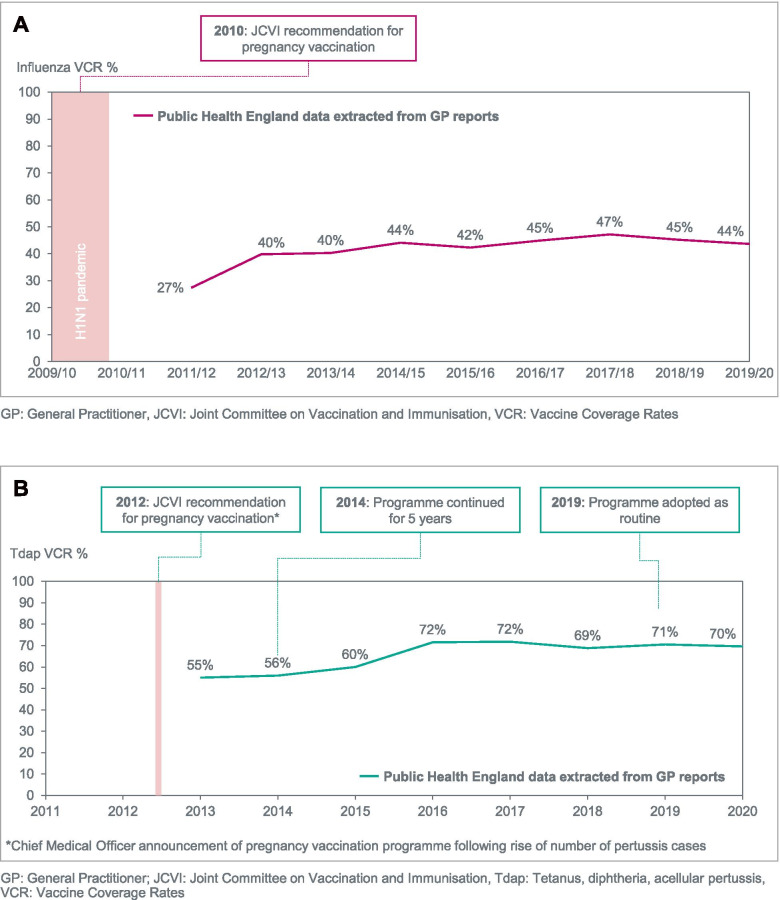
Evolution of VCR for influenza (2011–2020) and Tdap (2013–2020) among pregnant women in the UK. **A** Influenza. **B** Tetanus, diphtheria, acellular pertussis (Tdap)

**Fig. 3:**
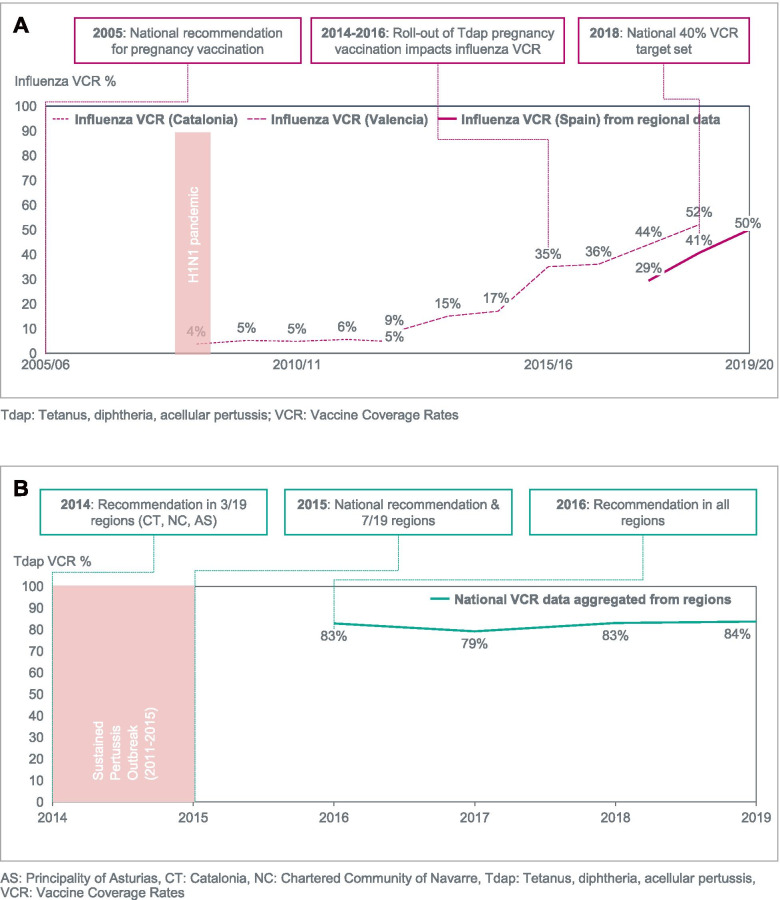
Evolution of VCR for influenza (2008–2020) and Tdap (2016–2019) among pregnant women in Spain. **A** Influenza. **B** Tetanus, diphtheria, acellular pertussis (Tdap)

The implementation of the pregnancy vaccination programmes in Spain, the UK, and the US has led to coverage rates ranging from 44% to 61% for influenza and from 57 to 84% for Tdap (Figs. 1, 2, 3). In each country, VCR growth rate has generally been slower for seasonal influenza vaccination than for Tdap, despite earlier healthy authority recommendations and funding of vaccination costs [36]. For pertussis vaccination, the VCR improved to levels above 50% over a two-to-three year period in each country (Figs. 1, 2, 3).

### The United States – seasonal influenza vaccination in pregnancy

Seasonal influenza VCR data was collected from nationwide survey data published in several Mortality and Morbidity Weekly Reports (MMWR) articles and from Kennedy et al. [29, 37, 38]. The US pioneered influenza vaccination in pregnancy with a recommendation for at-risk pregnant women dating back to 1966, which was updated by the Advisory Committee on Immunisation Practices (ACIP) to all pregnant women in their third trimester in 1995, and expanded to the second trimester in 1997 [39–41]. By the early 2000s the VCR reached 10 to 20% (Fig. 1). In 2004, the recommendation was updated to include all trimesters in accordance with WHO guidance [11, 42, 43]. Despite an endorsement by the American College of Obstetricians and Gynecologists (ACOG) in 2004, the VCR remained below 30% until 2007 [44]. The burden associated with the A/H1N1 2009 pandemic led to an increased mobilization among scientific societies, practitioners and lay public alike [45]. As a result, the VCR reached 50% by 2012 and over 60% in 2019. Nonetheless, current values fall short of the 80% target set by Healthy People 2020, an initiative that develops sets of objectives to improve the health of all Americans [46].

### The United States – pertussis (Tdap) vaccination in pregnancy

Tdap VCR was gathered from nationwide survey data from the CDC and MMWR articles [28, 29, 47]. A recommendation for the vaccination of at-risk pregnant women with Tdap was first issued in 2007 but the overall VCR remained close to 0% (Fig. 1). Following the 2010 pertussis outbreak in California, a recommendation was issued by the state to expand Tdap vaccination to both at-risk and healthy pregnant women [48]. This was followed by the ACIP which issued a recommendation in 2011 to vaccinate pregnant women with no prior history of Tdap vaccination, updated in 2012 to all pregnancies [49, 50]. This move was actively endorsed by scientific societies and key HCP associations such as the ACOG in 2013 [51]. Guidelines were cascaded to their members and the medical care organizations also followed suit by including Tdap vaccination in the medical protocol of pregnant women [52]. The impact on VCR was substantial, increasing from 9% to 50% within 2 years, and was also driven by the high public awareness at the time fed by communication from multiple stakeholders around pertussis prevention (Fig. 1). Since 2014 however the VCR measured by CDC surveys has plateaued at 55%, suggesting, as for influenza vaccination, persisting issues with regard to access to vaccination, HCP engagement and vaccine acceptance [29].

### The United Kingdom – seasonal influenza vaccination in pregnancy

In the UK, VCR data was collected from PHE (Public Health England) which extracts and aggregates data from General Practitioners (GPs) electronic reports [53]. PHE issued a recommendation for influenza vaccination during pregnancy in 2010, following a Joint Committee on Vaccination and Immunisation (JCVI) decision, in response to the severity of the A/H1N1pdm09 strain among pregnant women [54, 55]. This decision was corroborated by the cost-effectiveness of the intervention [56, 57]. PHE and the NHS (National Health Service) updated their guidance, training kits, and awareness materials accordingly to support GPs. As a result, VCR quickly reached 27% in the first season, 2011/2012, and 40% in the 2012/2013 seasons (Fig. 2). However, the limited vaccine effectiveness and persisting myths around the risks of vaccination have contributed to a plateau of the VCR at sub-optimal levels around 45% since the 2014/15 season, markedly below the WHO and EU target of 75% (Fig. 2).

### The United Kingdom – pertussis (Tdap) vaccination in pregnancy

For Tdap pregnancy vaccination, the VCR increase occurred faster due to high disease awareness among healthcare professionals and lay public alike. The national pertussis outbreak in 2012 caused the deaths of 14 non-immunized infants [58]. In a context of intense media noise on the large increase in cases and deaths reported by the Health Protection Agency (HPA), the JCVI adopted the recommendation for Tdap vaccination during all pregnancies [59–62]. A rapid roll-out of the guidance, training resources and awareness material for GPs led by PHE and the NHS followed, leading to a VCR above 50% in the first year [63] (Fig. 2). As this programme was proven to be safe, highly efficient, and cost-effective, it was extended for a further 5 years in 2014 [64]. The recommendation of the vaccination timing was expanded in 2016 to include weeks 16–32 of pregnancy and obstetricians, gynecologists, and midwives were increasingly mobilized in addition to the GPs. The programme was eventually adopted as routine in 2019 with VCR having reached a plateau around 70% [63, 65]. (Fig. 2).

### Spain – seasonal influenza vaccination in pregnancy

Health authorities in Spain included pregnant women in their second or third trimester in the risk groups for influenza vaccination as early as 2005 [66]. This was rapidly extended to all trimesters starting in 2006 in regions such as Catalonia [67, 68]. Yet, influenza VCR remained consistently low among pregnant women, at ~ 5%, as highlighted in several regional studies [67, 69] (Fig. 3). The roll-out of the Tdap pregnancy vaccination programme had a positive impact on influenza vaccination of pregnant women and synergies between the programmes led to a significant increase in influenza VCR, ultimately reaching the national VCR target of 40% for pregnant women in the 2018/2019 season [69–72] (Fig. 3). VCR data aggregated from regional reporting by the Ministry of Health since 2017/18 has shown a sharp increase [70, 73].

### Spain – pertussis (Tdap) vaccination in pregnancy

The rapid adoption of Tdap vaccination during pregnancy was driven by a public health response to the rising number of pertussis cases and deaths in infants below 3 months of age due to a “permanent outbreak situation” from 2011 to 2015 [74]. In light of the recommendation from the JCVI in 2012 in the UK, scientific societies including the Spanish Association of Paediatrics (AEP) played an important role in highlighting the burden of pertussis to national and regional health authorities [75, 76]. This led to the adoption of new recommendations by three pioneering regions (Catalonia, Asturias, and Navarra). By the end of 2016, all regions had introduced a Tdap pregnancy vaccination program. An analysis rapidly confirmed the effectiveness of the approach with the Red Nacional de Vigilancia Epidemiológica (RENAVE) highlighting a decreased incidence of pertussis cases in infants under 1 year of age and especially those under 3 months of age [74]. Since 2016, Tdap VCR data aggregated from regional reporting available from the Ministry of Health has been consistently at or above 80% since 2016 [70] (Fig. 3).

### The five-pillar VCR framework and adaptation of the list of components

As part of this analysis, a set of key components driving vaccine uptake was identified. The pre-existing five-pillar framework for influenza VCR was utilised to organise a list of components tailored to the specificities of seasonal influenza and Tdap vaccination in pregnancy [34]. The 5-pillar structure was confirmed as applicable and the pillars were adapted as follows:

1. Health Authority accountability and strengths of the pregnancy vaccination programme.
2. Facilitated patient access to vaccination.
3. Healthcare professional accountability and engagement.
4. Awareness of the burden and severity of diseases.
5. Belief in the benefits of pregnancy vaccination.

A total of 45 components were identified as relevant contributing factors to the successful roll-out of a pregnancy vaccination programme (Table 2). Of these, 15 components were unchanged from the previous framework, 14 were adapted, 16 were added as specific to the context of vaccination in pregnancy, and 13 removed as not applicable [34]. The national health authority recommendation, the absence of financial barriers to getting immunized, and a structured infectious disease surveillance network are applicable to maternal immunisation programme and were amongst the components that remained unchanged. The components that were adjusted to the context of vaccination in pregnancy include but are not limited to multiple HCPs (Ob-Gyns, GPs, midwives, nurses, pharmacists) being allowed to vaccinate, training of HCPs on pregnancy vaccination by multiple stakeholders, and adapted HCP training material for pregnancy vaccination. The new components specifically identified for the vaccination of pregnant women include the mention of immunization in the pregnant women medical protocol, a clear delineation of HCP roles and responsibilities, and the availability of adapted awareness material at maternal care providers.

**Table 2:** List of components among the five pillars contributing to high influenza and Tdap VCR among pregnant women

Pillar 1 Health authority (HA) accountability and strengths of the pregnancy vaccination programme	Pillar 2 Facilitated access to vaccination	Pillar 3 Healthcare professional (HCP) accountability and engagement	Pillar 4 Awareness of the burden and severity of diseases	Pillar 5 Belief in pregnancy vaccination benefits
HA leaders convinced of importance of pregnancy vaccination^b^	No financial barriers to getting immunized^c^	HCP associations actively endorsing pregnancy vaccination^b^	Structured infectious disease surveillance network^c^	Trust in vaccine safety during pregnancy^b^
Strong HA recommendation for pregnancy vaccination^b^	Access to multiple vaccination settings^c^	Clear delineation of HCP roles and responsibilities^a^	Well researched local pertussis outbreaks / burden^a^	Confidence in vaccine effectiveness of pregnancy vaccination^b^
Official HA recommendation systematically followed by full reimbursement^a^	Multiple HCPs (Ob-Gyns, General Practitioners, midwives, nurses) allowed to vaccinate^b^	Strong recommendation or referral from (legally) accountable HCPs^a^	Awareness of influenza / pertussis severity^b^	Proven evidence of the cost effectiveness of pregnancy vaccination programmes^a^
Immunisation in the pregnant women medical protocol^a^	Convenient and well-structured pregnancy patient journey^b^	Vaccination as part of routine activities in antenatal care^a^	HA on-site educational toolkit for pregnancy vaccination^a^	Trust towards HA and HCP communication
VCR targets at national, regional and health setting levels^c^	High frequency of antenatal visits (especially risky pregnancies)^a^	Training of HCPs on pregnancy vaccination by multiple stakeholders^b^	Mass media HA communication campaign^c^	Positive mass media coverage of pregnancy vaccination^b^
Accurate and regular VCR monitoring at vaccination site^c^	Immediate availability of vaccines for HCPs^c^	Adapted HCP training material for pregnancy vaccination^a^	Patient associations actively supporting pregnancy vaccination^b^	Knowledgeable KOLs vocal on pregnancy vaccination^a^
Proactive regional health authorities making pilots^c^	Vaccination patient reminders sent to pregnant women^b^	Fair and specific HCP compensation per vaccination^c^	Pregnancy vaccination stated on mummy blogs or by bloggers^a^	Limited trust & noise from “anti-vax” groups^c^
Accurate forecasting of volumes needed for pregnancy vaccination^a^	HCP pop-up notification to vaccinate pregnant women^b^	VCR-linked financial incentive or penalty for HCP^c^	Pregnant women convinced of medical need to protect their baby^a^	Monitoring and responsiveness vaccine disinformation^c^
Sustainable procurement system to ensure appropriate vaccine supply^c^		Immunisation status that can be checked across HCPs^c^		Attitudinal surveys on pregnant women and HCPs^a^
Efficient vaccine last-mile distribution system^c^		Acceptance or refusal in a signed consent form^a^		

^a^ New Components, ^b^ Adapted Components from Kassianos et al. (2021), ^c^ Unchanged Components from Kassianos et al. (2021) [77]

### Results of the five-pillar assessment across the three countries

**Fig. 4:**
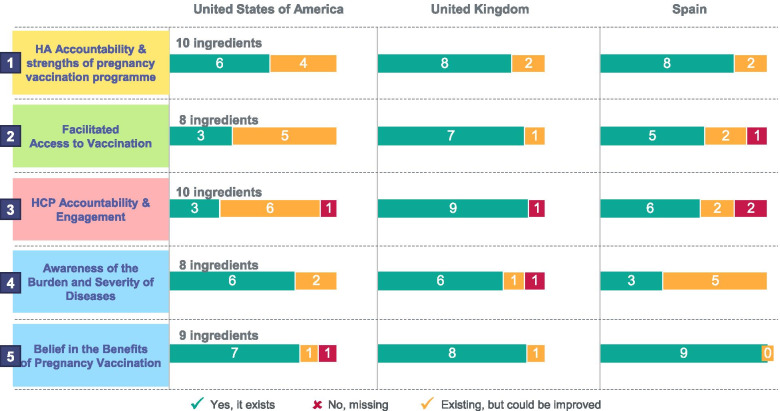
Pregnancy VCR gap analysis for the US, the UK and Spain

The adjusted five-pillar VCR framework of 45 components was used as a methodological basis to analyse the factors that contributed to the successful programmes in the US, the UK and Spain. There was significant variability across the three countries, which is likely to be attributable to the structural differences in the healthcare architecture and strategy applied for the commissioning of the vaccination programme in pregnancy. Overall, the UK had the highest number of components fully implemented (38 out of 45), followed by Spain (31 out of 45), and the US (25 out of 45). In the UK, five components were deemed as partially implemented, compared with 11 in Spain and 18 in the US. Finally, two components were missing in both the UK and the US, and three in Spain (Fig. 4). These differences mainly concerned components relating to the role of VCR targets and monitoring, the convenience of the patient journey, and the importance of the different HCPs involved in recommending and delivering vaccination in pregnancy. The three countries also differed in how adequate training was provided to HCPs as well as the incentivization for vaccinating pregnant women. (Additional file 1, Figs. SI 1–3) Finally, while the framework applied to both influenza and pertussis, some nuances between the two must be considered. The VCR of pertussis vaccination in pregnancy was higher than for influenza in all three countries, possibly because this programme emphasizes the indirect protection of the infant from the potentially serious complications of pertussis in infancy. Although there are benefits of influenza vaccination in pregnancy for infants, these may be less well-known or communicated to pregnant women. Finally, the seasonal nature of influenza vaccination has practical implications which must be considered for both the timing of vaccine administration and the identification of pregnant women.

## Discussion

The Pregnancy VCR framework developed in this study is designed to facilitate the implementation of vaccination programmes for pregnant women by providing a list of possible components structured in five pillars. The onus would remain with an individual country to determine the applicability and relevance of a specific intervention. The significant variability of the results indicates that having a successful programme does not rely on having all components but rather finding the right combinations of components that are tailored to each country’s context and healthcare system architecture.

The US, the UK, and more recently Spain, have monitored VCR following the introduction of their pregnancy vaccination programmes, enabling the analysis of the evolution of vaccine uptake and the components associated with VCR growth. The vaccination programmes analysed owe a large part of their success to proactive health authorities (Pillar 1). Beyond establishing an official vaccination recommendation and reimbursement, supporting programme implementation is critical. In the UK, PHE and the NHS were instrumental in producing HCP training material and information for patients when commissioning the immunisation programme [78]. In Spain, the proactive development of such communication materials by some regional health authorities is likely to have contributed to the higher performance of some regions such as *Cantabria* and *Comunitat Valenciana*, both achieving Tdap VCR above 90% among pregnant women [26, 66]. In the US, given the higher diversity of stakeholders, a multi-stakeholder approach with frequent and coordinated calls to action from the CDC and the leading scientific societies were essential to ensure the continued uptake of pregnancy vaccinations. For instance, the 2020 maternal task force reinforced the importance of a one-voice message from key HCP associations involved in the care of pregnant women [79, 80]. Another key lever that health authorities can act upon is to include pregnancy vaccination as part of the routine antenatal medical protocol [81]. Furthermore, continuous and accurate monitoring of VCR is a key element for the evaluation of a programme after its introduction and can be complemented by setting official VCR targets.

Facilitated access to vaccination (Pillar 2) with minimum physical, geographic, and financial barriers stands out as critical. Beyond offering vaccination free of charge, providing pregnant women with the opportunity to be vaccinated in convenient settings including GP clinics, community antenatal clinics, hospitals, or pharmacies, and authorising multiple HCP roles to vaccinate pregnant women was key. In the US, access to influenza vaccination is generally convenient, and pregnant women are often immunised by obstetricians, gynecologists, or pharmacists [28]. In the UK and Spain, midwives accompany women throughout their pregnancy and therefore play a decisive role in driving awareness of the recommendations for vaccination [72, 82, 83]. A study in the Greater Manchester area in the UK highlighted the beneficial impact of coordinating GP surgeries with antenatal services, pharmacists, NHS foundation teams, and primary care trusts to maximize uptake among pregnant women. An effective electronic vaccine record and the allocation of adequate training resources to align messages across HCPs were found to be important components [84]. Initiatives such as *text4baby* in the US have also proved to be efficient calls to action to prompt pregnant women to get vaccinated [85, 86].

Regarding the accountability and engagement of HCPs (Pillar 3), a strong recommendation from an HCP is a clear driver of vaccine uptake for pregnant women as shown in multiple surveys from the US CDC [28, 38]. The mobilization of antenatal care professionals, such as obstetricians, gynecologists, and midwives is essential, given the importance of their advice for pregnant women and the multiple medical touchpoints during pregnancy. As such, in the US, the active endorsement of the ACIP recommendation by multiple HCP societies was crucial for the adoption of Tdap pregnancy vaccination [51]. Additionally, specific training and toolkits are produced and disseminated by the CDC and the ACOG to encourage obstetricians and gynecologists to vaccinate pregnant women [87–89]. In the UK, influenza and Tdap pregnancy vaccination is fully part of the contractual agreement between the health authorities and GPs, and includes the financial incentivization of vaccination in pregnancy (£10.06 per immunisation in 2019) as compensation for the obligation to record immunisation status, call and recall eligible individuals, and document active refusals [90].

Finally, awareness of the disease burden and belief in vaccination benefits are paramount to a successful programme (Pillars 4 and 5). The awareness of the burden of the disease relies on a well-established infectious disease surveillance network, whose epidemiological and clinical data can be used for research, public health decision making, and communication purposes [82]. The knowledge of the disease severity and vulnerability to the infection have an immediate effect on VCR as illustrated by the impact of the pertussis outbreaks in the UK and Spain at the inception of their respective programmes [59, 75, 76]. In the US, patient associations such as the California Immunisation Coalition have developed collections of stories to illustrate the burden of vaccine-preventable diseases such as pertussis. Confidence in vaccine safety and vaccine effectiveness can nonetheless vary widely, especially for vaccination in pregnancy, depending on cultural, ethnic, and socioeconomic factors [77]. An active monitoring and responsiveness towards vaccine disinformation complemented by regular attitudinal surveys of the perceptions of both pregnant women and the HCPs who take care of them are essential to address misconceptions and tailor communication strategies [91–94].

While the benefits of pregnancy vaccination programmes are acknowledged by the public health community, increasing VCR remains a complex challenge which can often present an added level of difficulty for the vaccination of pregnant women given the persisting misconceptions on the safety of such interventions [36, 52, 95]. A large number of studies have gathered quantitative insights on the knowledge, attitudes, and practices of pregnant women and HCPs with the aim to suggest avenues for improvement [36, 69, 92, 96, 97]. However, few studies have isolated programmatic and policy directed interventions at the initiation of programmes that lead to high pregnancy vaccination uptakes for influenza or for Tdap [14, 98]. No previous studies have documented the experience of successful pregnancy vaccination programmes for influenza and pertussis across countries with different healthcare systems.

As such, this is the first study to describe the components of a successful pregnancy programme for both influenza and Tdap vaccinations across all the different stakeholders involved. These components are clustered in 5 pillars, providing a ready-to-use framework to complement the field guide developed by the WHO for Latin America and the WHO manual on the implementation of influenza vaccination of pregnant women [30, 99].

This analysis also provides a comprehensive framework to perform a situation assessment and identify policy and programmatic gaps hindering high VCR in pregnancy. Given the increasing number of countries issuing recommendations for vaccinating pregnant women, and the room for improvement in several existing programmes, including the three countries covered in this study, this analysis of best-in-class practices highlights key drivers for uptake. In the current context of the COVID-19 pandemic, some of the key learnings from this study could also be useful for scientific discussions and policy-making regarding COVID-19 VCR in pregnant women [77].

This study has several limitations. Firstly, the selection of the studied countries could have been widened to include other countries with well-established pregnancy vaccination programmes. For instance, surveys have been performed in Canada and Australia with the objective of identifying challenges regarding vaccination during pregnancy and strategies to overcome these issues [100, 101]. Moreover, the three countries are examined from national perspective that does not take into account nuances and disparities across their regions or states. VCR estimation methods also vary from country to country with CDC data based on surveys for the US, while PHE data is based on GP reports, and Spanish data from reporting in each region. Furthermore, the adaptability of the framework to low- or middle-income countries is limited since only high-income countries were selected for this analysis. High-performing pregnancy vaccination programmes also exist in upper middle income countries such as Argentina and Mexico [102–104].

## Conclusions

This study has shown that the components of successful pregnancy vaccination programmes can be analysed through five key pillars for success. Within each of these pillars a list of components that combine to drive vaccine uptake was identified, with the US, the UK and Spain each having their own specific combination of components contributing to high VCRs among pregnant women. The three studied countries show that a successful VCR does not necessarily require the implementation of all components; instead, the combination of components should be fine-tuned according to the specificities of the healthcare system in place and in consideration of the societal and cultural aspects of pregnancy vaccination. This framework can therefore serve as a guiding tool for public health experts, health authorities, and HCPs to identify the most relevant components for the successful implementation of a pregnancy vaccination programme that can be adapted to the local context.

## Supplementary Information

**Additional file 1.**

## Data Availability

All data generated or analysed during this study are included in this published article and its supplementary information files.

## Abbreviations

ACIP: Advisory Committee on Immunisation Practices
ACOG: American College of Obstetricians and Gynecologists
AEP: Spanish Association of Paediatrics
CDC: Centers for Disease Control and Prevention
GMS: General Medical Service
GPs: General Practitioners
HA: Health authority
HCP: Healthcare professional
HPA: Health Protection Agency
JCVI: Joint Committee on Vaccination and Immunisation
KOLs: Key opinion leaders
NHS: National Health Service
PHE: Public Health England
RENAVE: Red Nacional de Vigilancia Epidemiológica
Tdap: Tetanus, diphtheria, acellular pertussis
UK: United Kingdom
US: United States
VCRs: Vaccine coverage rates
WHO: World Health Organization
