# Supplementary material for: Vaccination in pregnancy against pertussis and seasonal influenza: key learnings and components from high-performing vaccine programmes in three countries: the United Kingdom, the United States and Spain
Source: BMC Public Health. 2021 Nov 29;21:2182. doi: 10.1186/s12889-021-12198-2 (PMC8628032; doi:10.1186/s12889-021-12198-2)

**ADDITIONAL FILES**

**Vaccination in pregnancy against pertussis and seasonal influenza: Key learnings and components from high-performing vaccine programmes in three countries: the United Kingdom, the United States and Spain**

**ADDITIONAL FILE 1**

**Table SI 1:** Search terms used for the scientific literature review on Embase

| **Disease** | **Vaccination** | **Population** |
| --- | --- | --- |
| Pertussis | Vaccination | Pregnant women |
| Tetanus | Immunisation | Pregnancy |
| Diphtheria | Vaccine uptake |  |
| Influenza | Vaccine Coverage |  |
|  | Drivers |  |
|  | Barriers |  |
|  | Incidence |  |
|  | Knowledge |  |
|  | Attitudes |  |
|  | Perceptions |  |
|  | Determinants |  |
|  | Predictors |  |
|  | Factors |  |
|  | Reasons |  |

**Table SI 2:** PICO process used for the scientific literature review on Embase

| **PICO** | **Inclusion Criteria** | **Exclusion Criteria** |
| --- | --- | --- |
| Patient | - Pregnant women | - Other population groups |
| Intervention | - Seasonal influenza vaccination during pregnancy - Tdap vaccination during pregnancy | - Other vaccinations |
| Comparison | - Any - None | - N/A |
| Outcome | - Vaccine Coverage Rates - Vaccine Knowledge & Attitudes - Vaccine Barriers & Drivers | - Vaccine efficacy - Vaccine effectiveness |

PICO: Patient, Intervention, Comparison, Outcome ; N/A : Non Applicable ; Tdap : Tetanus, Diphtheria, acellular Pertussis

**Figure SI 1:** Pregnancy Vaccine Coverage Rates (VCR) components for the US based on the 5 pillars of the Pregnancy VCR framework


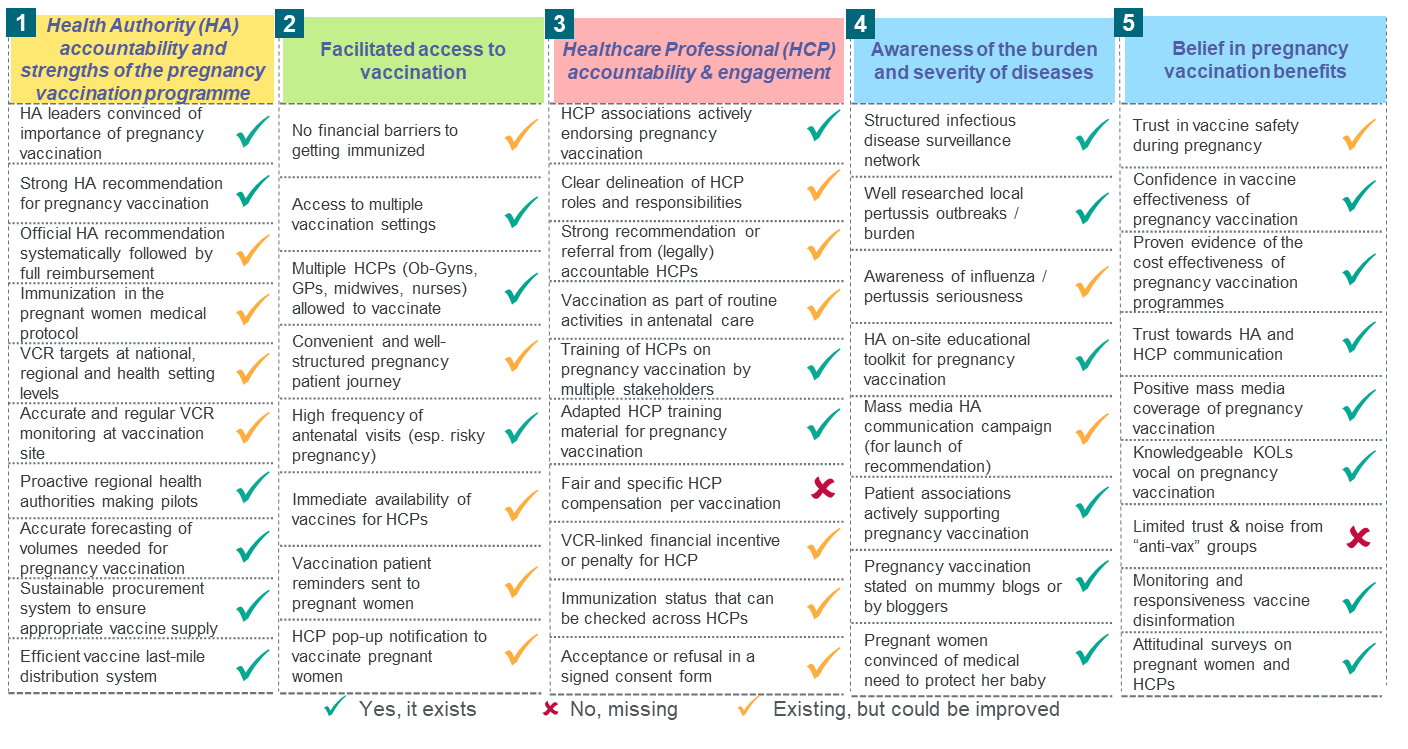


**Figure SI 2:** Pregnancy Vaccine Coverage Rates (VCR) components for the UK based on the 5 pillars of the Pregnancy VCR framework


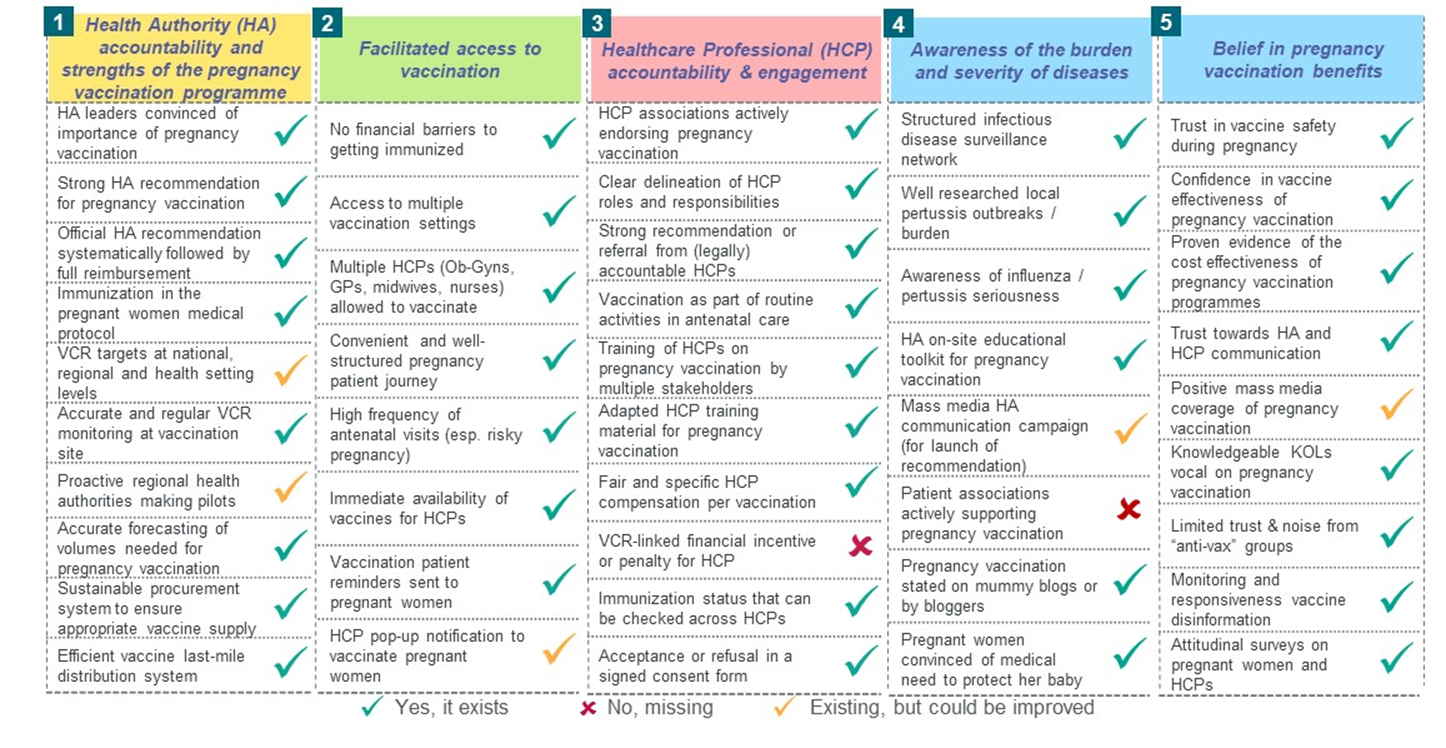


**Figure SI 3:** Pregnancy Vaccine Coverage Rates (VCR) components for Spain based on the 5 pillars of the Pregnancy VCR framework


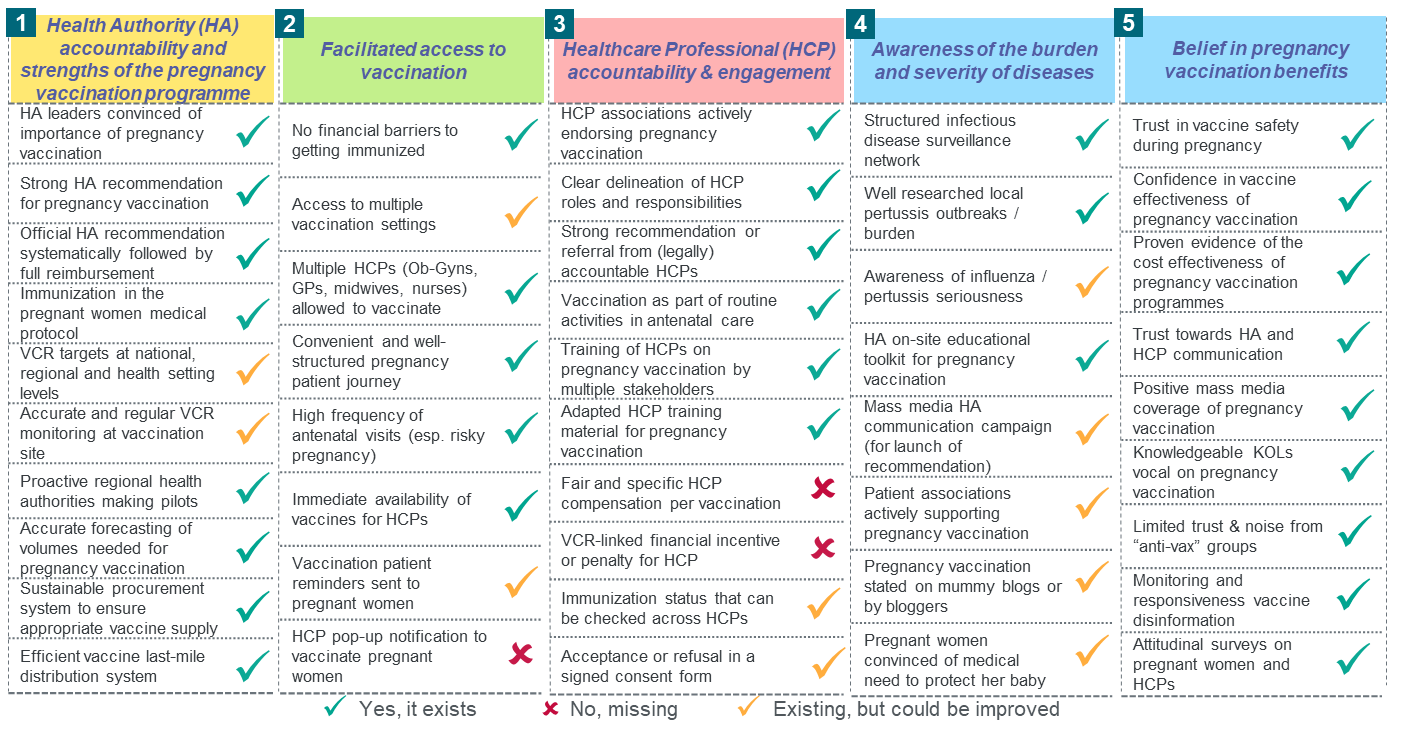

Supplement: Supplementary file 1 — Additional file 1. [file 12889_2021_12198_MOESM1_ESM.docx]
